# Collagen External Scaffolds Mitigate Intimal Hyperplasia and Improve Remodeling of Vein Grafts in a Rabbit Arteriovenous Graft Model

**DOI:** 10.1155/2017/7473437

**Published:** 2017-04-19

**Authors:** Haiming Li, Shoudong Chai, Longsheng Dai, Chengxiong Gu

**Affiliations:** ^1^Department of Cardiac Surgery, Beijing Anzhen Hospital, Capital Medical University, Beijing, China; ^2^Beijing Institute of Heart Lung and Blood Vessel Diseases, Beijing, China

## Abstract

*Objectives*. The aim of this study was to test the effects of collagen external scaffold (CES) in intimal hyperplasia of vein grafts and explore its underlying mechanisms.* Methods*. Thirty-six New Zealand white rabbits were randomized into no-graft group, graft group, and CES group. The rabbit arteriovenous graft model was established. In CES group, the vein graft was wrapped around with CES. The hemodynamic parameters of vein grafts were measured intraoperatively and 4 weeks after operation by ultrasonic examination. Histological characteristics of vein grafts were also evaluated 4 weeks later. The mRNA and protein levels of proliferating cell nuclear antigen (PCNA), active cleaved-caspase-3 (ClvCasp-3), and smooth muscle 22 alpha (SM22*α*) were measured 4 weeks later by quantitative real-time PCR and western blot.* Results*. CES significantly improved the hemodynamic stability of vein grafts, with higher blood velocity and blood flow. Similarly, CES also markedly mitigated intimal hyperplasia and inhibited dilatation of vein grafts. In CES group, the upexpression of PCNA and ClvCasp-3 and the downexpression of SM22*α* were inhibited.* Conclusion*. CES exerts beneficial effects in mitigating intimal hyperplasia and improving remodeling of autogenous vein grafts, which may be associated with reducing the proliferation and apoptosis and preserving the phenotype of VSMCs.

## 1. Introduction

Approximately one million coronary artery bypass grafting (CABG) and peripheral vascular reconstruction procedures are performed around the world each year, and the autologous saphenous vein graft remains the most popular conduit [1]. After implantation, the vein grafts undergo necessary “arterialization” to adapt to the arterial circulation. Along with the remodeling, the vessel wall becomes thicker due to the proliferation of vascular smooth muscle cells (VSMCs) and the deposition of extracellular matrix, both of which contribute to intimal hyperplasia (IH) and vein graft failure [2]. Uncontrolled IH may lead to vein graft failure and serious clinical complications. It has been reported that the rate of vein graft failure at 1 and 10 years is approximately 15%–20% and 50%–70%, respectively [3, 4]. Vein graft failure is not only devastating to individual patients but also results in huge healthcare costs.

Several diverse treatments, such as antiplatelet therapy [5], immunosuppressive therapy [6], and gene therapy [2, 7, 8], have been explored to inhibit IH to reduce the rate of vein graft failure in numerous research. However, the efficacies of those treatments have not been confirmed yet [4, 7, 9]. Accumulating evidence shows that the external scaffold is an effective surgical intervention to inhibit IH of vein graft in multiple experimental and clinical researches [10, 11]. Study on biodegradable stent brings a new direction for our research. Thus, biodegradable material seems to be much more appropriate in making external scaffold [12], because it not only plays a protective role in the early phase but also is helpful in reducing infection and foreign body reaction caused by the long-term existence of external scaffold. However, most of the external support materials used in these studies are synthetic, such as propylene glycol alginate and polylactic-co-glycolic acid, which have the problems of poor biocompatibility, low flexibility, and degradation [10]. Accordingly, it is very important to identify an appropriate biodegradable material.

Collagen provides physical support to tissues and plays a crucial role in maintaining biological and structural integrity of the extracellular matrix [13]. More importantly, the morphology, adhesion, migration, and differentiation of cells could be regulated by collagen because it features low immunogenicity and good biocompatibility, porous structure, and good permeability and biodegradability [14, 15]. In virtue of these excellent properties, it has been widely used in tissue engineering and has made great achievements [13]. However, collagen external scaffolds (CES), as a perivenous support for vein grafts, have not been reported to our knowledge. Thus, in this study, we tested the effects of CES in IH of vein grafts and explore its underlying mechanisms in a rabbit arteriovenous graft model.

## 2. Materials and Methods

The experimental and animal care protocol was approved by the Institutional Ethics Committee for Animal Care and Usage of Beijing An Zhen Hospital of Capital Medical University (ID: AEEI-2015-144).

### 2.1. Collagen External Scaffold

The collagen patch (40 × 20 × 0.5 mm) was provided by Shandong Academy of Pharmaceutical Science (Jinan, China). In the process of wrapping vein grafts, we sutured the long side of the collagen patch in interrupted fashion using 8-0 Prolene suture to form a tubular external scaffold with an internal diameter of approximately 4.5–5.0 mm.

### 2.2. Study Design

**Figure 1:**
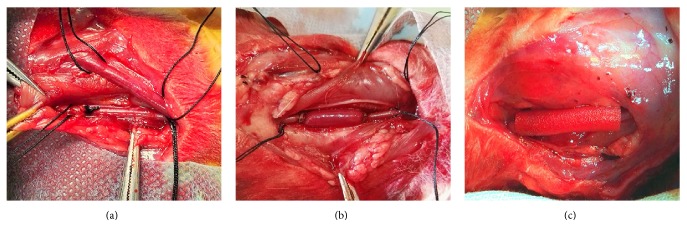
Experimental grouping. No-graft group (a), graft group (b), and CES group (c). *N* = 12 in each group.

Thirty-six New Zealand white male rabbits weighing 2.0–2.5 kg (Beijing Fang Yuan Farm, Beijing, China) were randomized into three groups: no-graft group (only free of jugular vein and carotid artery, *n* = 12, Figure 1(a)), graft group (interposing reversed jugular vein graft into the carotid artery without CES placement, *n* = 12, Figure 1(b)), and CES group (arteriovenous graft with CES placement, *n* = 12, Figure 1(c)).

### 2.3. Surgical Methodology

The rabbits were anesthetized by abdominal injection (1 ml/kg) and ear side vein injection (0.5 ml/kg) of 3% pentobarbital sodium. Vital signs were closely observed during the operation. If the rabbit developed apnea, mechanical ventilation was then constructed (DW-3000B; Beijing Zhongshidi Science and Technology Development Co., Ltd, Beijing, China). Next, heparin (500 IU/kg) was intravenously injected via the ear vein. The surgical area was disinfected and locally anesthetized by subcutaneous injection of 2.5 ml of 2% lidocaine. The external jugular vein in the superficial fascia was fully dissociated using the “no-touch” approach after the skin was incised under aseptic conditions. Bulldog hemostatic clamps were placed on the proximal and distal ends of the vein and artery. Then, the external jugular veins (3.0–4.0 cm) were harvested after ligation of the vein ends, and a section of carotid artery (2.0–3.0 cm) was removed. The isolated external jugular vein was anastomosed to the carotid artery reversely by using a vascular anastomosis stapler wheel (1.5 mm; Shandong Xinhua Surgical Instruments Co., Ltd, Zibo, China). After the anastomosis, hemostatic clamps at the carotid artery were released, and the quality of the anastomosis was examined carefully. For rabbits in the CES group, the tubular collagen external scaffold was placed around the entire external jugular graft, including both anastomotic sites. After careful examination, the incision was closed layer by layer. Finally, the diameter, blood velocity, and blood flow of the vein grafts were measured. Penicillin (4 million IU) was administered by intramuscular injection for 5 days, and oral aspirin (50 mg) was administered for 7 days.

Surgery was performed again 4 weeks after the operation. After applying the same anesthesia, the original incision was reopened. The vein grafts, including bilateral native carotid arteries, and normal external jugular veins were cut off. Then, the sample was evenly divided into five parts. Three parts taken from the proximal, middle, and distal part of the sample were fixed in 4% paraformaldehyde and then were embedded in paraffin. The remaining two parts were cooled by liquid nitrogen and then stored at −80°C. After drawing, those rabbits were kept feeding.

### 2.4. Vascular Ultrasound Examination

The diameter, blood velocity, and blood flow of vein grafts were measured intraoperatively and 4 weeks after the operation using the Vevo 2100 Imaging System (VisualSonics Inc., Canada). All measurements were performed three times, and the average was recorded. The change rate of above-mentioned indexes was calculated according to the description of the previous study [16]. The change rate of the peak velocity was determined as (*V*1 −* V*2)/*V*1 (*V*1 denotes the velocity right after the interposition;* V*2 denotes the velocity at 4 weeks postoperatively). The change rate of peak flow is similar to the above-mentioned calculation method. But, the change rate of the diameter was calculated as (*D*2 −* D*1)/*D*1 (*D*1 denotes the primary diameter;* D*2 denotes the diameter at 4 weeks postoperatively).

### 2.5. Histological Examination

The samples fixed in 4% paraformaldehyde were dehydrated, embedded in paraffin, and transected into 4 *μ*m sections. Next, the sections were stained with hematoxylin and eosin (HE) and Masson's trichrome stain to examine the structural change of vein grafts. Three slices were taken from each rabbit, which came from the proximal, middle, and distal part of the sample, respectively. The intima and media thickness were measured using a computer image analysis system (NIKON NIS-Element imaging system, Shanghai, China), with each specimen measured three times and the average was computed.

### 2.6. Immunohistochemical Staining

Part slices were stained using a two-step immunohistochemical staining technique. The immunohistochemical staining used the following primary antibody: mouse monoclonal against proliferating cell nuclear antigen (PCNA, Abcam) and rabbit polyclonal against active cleaved-caspase-3 (ClvCasp-3, Abcam) at dilutions 1 : 500 and 1 : 1000, respectively. Staining images (40x objective lens) were acquired and analyzed using the NIKON NIS-Element imaging system. Brown-stained cells in both the intima and media were considered positive.

### 2.7. Western Blotting

Western blotting (WB) was used to analyze the protein levels of PCNA, ClvCasp-3, and smooth muscle 22 alpha (SM22*α*), using the method as previously reported [17]. In addition to the previously mentioned two primary antibodies, goat polyclonal against SM22*α* (Abcam) was also used at dilutions 1 : 1000. To evaluate the total protein levels, the membranes were probed with a mouse monoclonal against *β*-actin (ImmunoWay), diluted 1 : 5000.

### 2.8. Quantitative Real-Time Polymerase Chain Reaction

Quantitative real-time polymerase chain reaction (PCR) analysis was performed on vein mRNA as previously described [18], using the primers given in Table 1. Glyceraldehyde-3-phosphate dehydrogenase (GAPDH) was used as the housekeeping gene. The relative mRNA levels of the target gene to the housekeeping gene were calculated as 2^(CT_GAPDH_ − CT_target_)^.

### 2.9. Statistical Analysis

All data was analyzed using the statistical analysis software SPSS 17.0. The data was presented as the mean ± standard deviation. Comparison among multiple groups was analyzed by one-way analysis of variance (ANOVA). Bonferroni method was also performed for two group comparisons. *P* value < 0.05 was considered statistically significant.

## 3. Results

### 3.1. The Hemodynamic Stability of the Vein Graft

**Figure 2:**
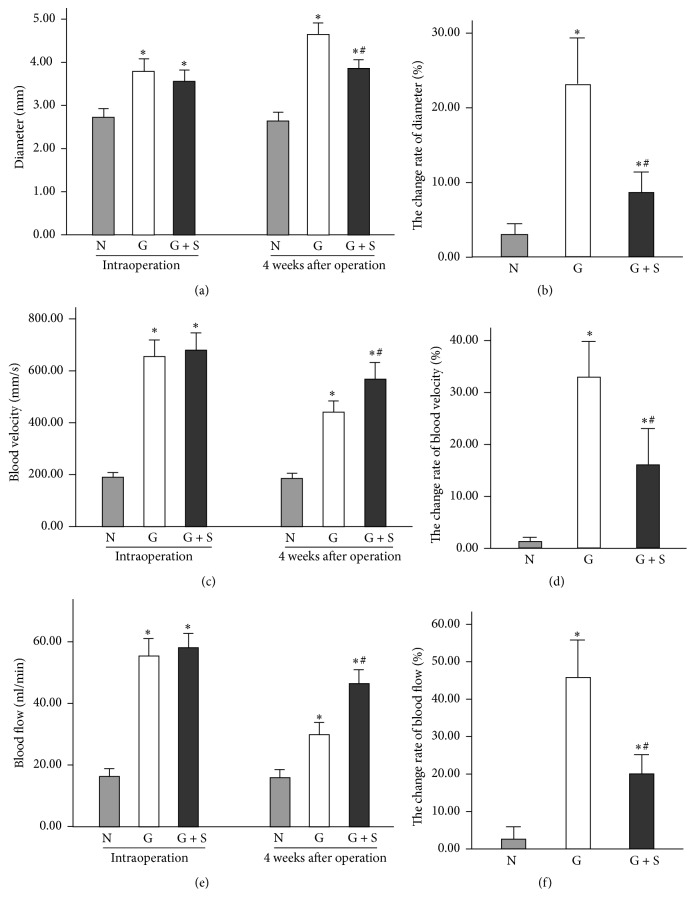
CES markedly improves the hemodynamic stability of the vein graft. (a–f) Columnar diagrams show the results of vascular ultrasound examination. The CES effectively inhibited the expansion and prevented the reduction of the blood velocity and flow of the vein graft. N: no-graft group; G: graft group; G + S: CES group. ^*∗*^*P* < 0.05 versus no-graft group; ^#^*P* < 0.05 versus graft group.

All rabbits survived, and all of the vein grafts were unobstructed 4 weeks later. The intraoperative diameter of the graft group was similar to that of the CES group, but the diameter of the no-graft vein was smaller than that of the graft group and CES group (Figure 2(a)). Four weeks later, the diameter of the graft group was obviously larger than that of the CES group (4.64 ± 0.27 versus 3.85 ± 0.21 mm, *P* < 0.05; Figure 2(a)). Thus, the change rate of the diameter of the graft group was larger than that of the other two groups (Figure 2(b)), indicating that the CES could effectively inhibit the expansion of the vein grafts. The intraoperative blood velocity of the graft group was similar to that of the CES group, but there was a significant difference between the no-graft group and the graft group (Figure 2(c)). Interestingly, the blood velocity of the graft group significantly decreased 4 weeks later, whereas the blood velocity of the CES group was markedly higher than that of the graft group (565.68 ± 63.77 versus 438.05 ± 44.24 mm/s, *P* < 0.05; Figure 2(c)). In addition, the change rate of the blood velocity in the graft group was larger than that in the CES group (Figure 2(d)). Thus, the dropped blood velocity of vein graft could be also prevented by the CES. Consistent with the graft blood velocity, the blood flow was significantly lower in the graft group compared with the CES group 4 weeks after the operation (29.80 ± 3.87 versus 46.27 ± 4.50 ml/min, *P* < 0.05; Figure 2(e)), although there was no significant difference intraoperatively. Additionally, the change rate of blood flow in the graft group was also larger than that in the CES group (Figure 2(f)), suggesting that the CES significantly prevented the reduction of blood flow of graft vein.

### 3.2. The Intimal Hyperplasia and Remodeling of the Vein Graft

**Figure 3:**
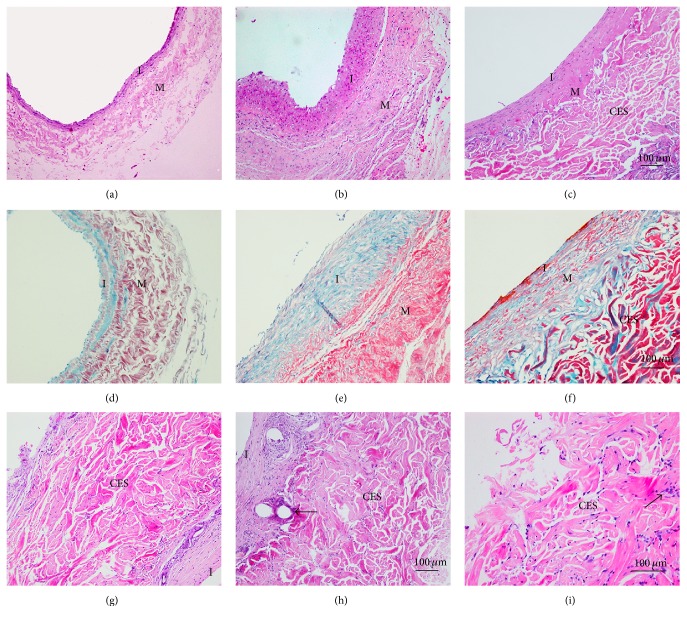
CES reduces the thickening of intimal and improves the remodeling of the vein graft. (a–c) Sections stained by HE of the normal vein (a), vein grafts from the graft (b), and CES (c) groups. (d–f) Collagen deposition (blue) was seen in vein grafts (e) and was largely decreased by the CES (f) in the sections stained by Masson. (g–i) Sections stained by HE of the CES showed fusion of the CES and adventitia of the vein graft (g), formation of vasa vasorum (h), degradation of the CES, and infiltration of VSMCs in CES (i). I: intima; M: media. The arrows, respectively, indicate the formation of vasa vasorum in the space between CES and vein graft (h) and the infiltration of VSMCs (i).

**Figure 4:**
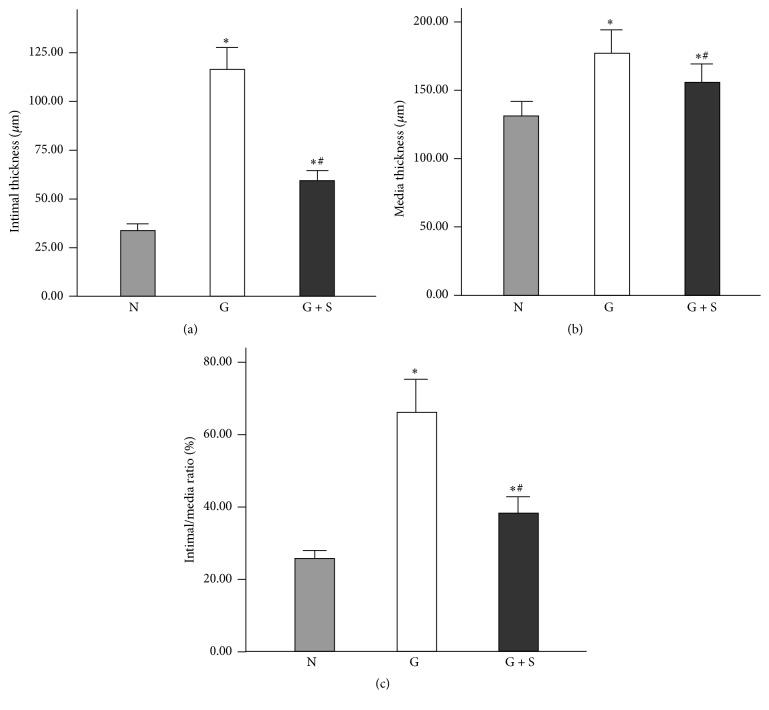
Columnar diagrams show the structural changes of vein grafts. N: no-graft group, G: graft group, and G + S: collagen external scaffold group. ^*∗*^*P* < 0.05 versus no-graft group; ^#^*P* < 0.05 versus graft group.

Sections stained with HE (Figures 3(a)–3(c)) showed that the intima and media of the graft group were dramatically thicker than those of the normal vein (116.67 ± 10.82 *μ*m versus 33.81 ± 3.37 *μ*m and 177.33 ± 15.96 *μ*m versus 131.25 ± 10.06 *μ*m, respectively, *P* < 0.05; Figures 4(a) and 4(b)). However, the CES group exhibited substantially thinner intima and media than the graft group (59.48 ± 5.17 *μ*m versus 116.67 ± 10.82 *μ*m and 155.89 ± 13.05 *μ*m versus 177.33 ± 15.96 *μ*m, respectively, *P* < 0.05; Figures 4(a) and 4(b)). For the intima/media ratio, the CES group, similar to the no-graft group, was significantly lower than that of the graft group (38.43 ± 4.66% versus 66.33 ± 9.16%, *P* < 0.05, Figure 4(c)). Sections stained with Masson showed that collagen was specifically displayed in the wall of normal vein (Figure 3(d)). However, much more collagen arranged in disorder was detected in the intima and media of the vein graft in the graft group (Figure 3(e)). Besides, less collagen well arranged in the intima and media was found in the CES group (Figure 3(f)). Furthermore, sections stained by HE of the CES demonstrated perfect fusion between the CES and the adventitia of vein grafts (Figure 3(g)), formation of vasa vasorum in the space between CES and vein grafts (Figure 3(h)), slow degradation of the CES, and infiltration of VSMCs (Figure 3(i)).

### 3.3. The Proliferation, Apoptosis, and Phenotypic Transition of VSMCs

**Figure 5:**
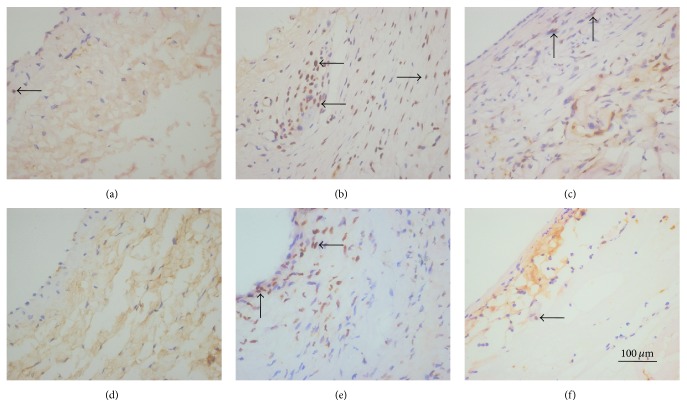
Immunostaining revealed PCNA (a–c) and ClvCasp-3 (d–f) in no-graft veins and a sizable increase of the staining (brown, arrow) in graft veins ((a–c) and (d–f) represent no-graft group, graft group, and collagen external scaffold group, respectively).

**Figure 6:**
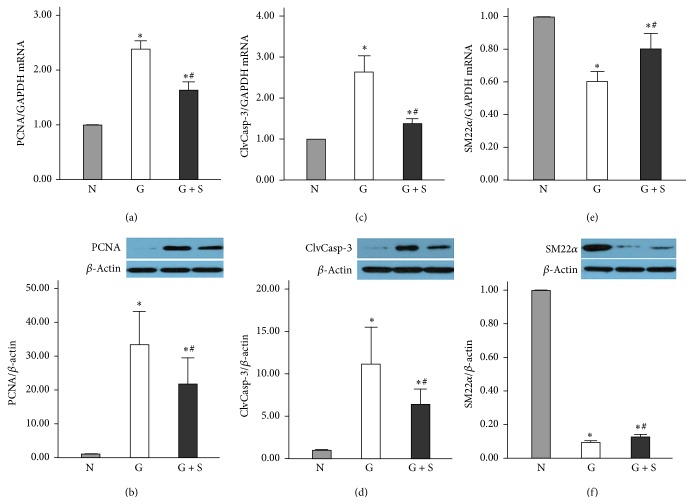
CES prevents the upregulation of PCNA and ClvCasp-3 and partly inhibits the downregulation of SM22*α*. (a and c) Compared with the no-graft group level, the PCNA and ClvCasp-3 transcripts of graft group were increased, and they were prevented in the presence of the CES. (e) The SM22*α* transcript of graft group and CES group decreased compared to the no-graft group, but those of CES were higher than those of graft group. (b and d) The PCNA and ClvCasp-3 proteins were abundantly expressed in graft veins, which were prevented by the CES. (f) The SM22*α* protein was significantly downregulated in graft group. However, the CES partly prevented the downregulation of SM22*α*. N: no-graft group; G: graft group; G + S: CES group. ^*∗*^*P* < 0.05 versus no-graft group; ^#^*P* < 0.05 versus graft group.

To further investigate the underlying mechanisms, we examined the changes of PCNA, ClvCasp-3, and SM22*α* to observe the proliferation, apoptosis, and phenotypic transition of VSMCs. Immunostaining revealed that there was a sizable increase in PCNA (Figures 5(a)–5(c)) and ClvCasp-3 (Figures 5(d)–5(f)) in the graft veins compared with that in the no-graft veins. In the graft group, there was a significant increase in the mRNA transcript and protein expression levels of PCNA compared with the no-graft veins that were prevented in the presence of the CES (1.64 ± 0.15 versus 2.40 ± 0.14 and 21.66 ± 7.77 versus 33.40 ± 9.88, respectively, *P* < 0.05; Figures 6(a) and 6(b)). Similarly, both the ClvCasp-3 transcript and protein levels were elevated in the graft veins. But, the CES reinforcement markedly prevented the increase in the ClvCasp-3 transcript and protein expression (1.37 ± 0.12 versus 2.64 ± 0.15 and 6.38 ± 1.81 versus 11.16 ± 4.35, respectively, *P* < 0.05; Figures 6(c) and 6(d)). Although there was a dramatic reduction in the transcription and expression of SM22*α* in the graft veins, the CES also partly inhibited its downregulations (0.80 ± 0.09 versus 0.60 ± 0.06 and 0.13 ± 0.01 versus 0.09 ± 0.01, respectively, *P* < 0.05; Figures 6(e) and 6(f)).

## 4. Discussion

In the present study, we have found that wrapping the graft veins with a tubular collagen scaffold could significantly inhibit the development of IH and improve the remodeling of the vein graft. Additionally, we have further revealed that the structural alterations were associated with changes in the proliferation, apoptosis, and phenotypic transition of VSMCs and in the expression of various genes and proteins, suggesting that the CES caused significant protection against these changes.

The structural characteristics of the vein graft make it prone to IH after transplantation into the arterial environment [19]. Many researchers used collagen to create tissue engineered blood vessels to replace vein grafts, but the tissue engineered grafts are likely to have thrombosis after implantation because of lacking normal endothelium structure [13]. Thus, in the current study, we wrapped the CES around the graft vein, mimicking arterial medial structure, to make it more like an artery in terms of the structure. Several fundamental experiments and clinical studies have already demonstrated that external support could efficiently prevent the dilation of venous walls and inhibit IH [10–12, 16, 18, 20, 21]. The external stent material used in previous experiments was easy to cause inflammation, foreign body reaction, and even fibrosis [22], which may be consistent with IH process. Because of the good biocompatibility, high permeability, and favorable biodegradability, collagen has been widely used in tissue engineering [13–15]. However, as far as we know, this is the first study in which collagen was used as a perivascular scaffold. Here, we showed that the novel CES was also efficient in preventing IH and improving the remodeling of the vein graft in a rabbit arteriovenous graft model. Interestingly, this study demonstrated that the CES and part of the adventitia of the vein grafts could be perfectly fused, indicating that the CES could provide sufficient mechanical support to inhibit the expansion, reduce turbulent flow, and improve the hemodynamic stability of the vein graft [10]. In addition, this study showed the formation of the vasa vasorum in the space between the CES and the vein grafts, suggesting that oxygen and nutrients could be supplied for the vein graft, and hypoxia-induced pathological changes could be alleviated [11]. We also found the infiltration of VSMCs in the CES with degradation, implying that it has become a “living” tissue and “real” wall structure of the vein graft. We conclude that, by meaningful reinforcement of the structure of the vein graft, the CES provides sufficient mechanical support and promotes the formation of the vasa vasorum, combining the advantages of constrictive and loose-fitting stents [10].

Our research also supplied cellular and molecular evidence regarding how the mechanical effects of the CES were translated into protective effects. The changes in the vein grafts were associated with the proliferation and apoptosis of VSMCs within the intima-media layer [18, 23]. Additionally, the imbalance of the proliferation and apoptosis of VSMCs played an important role in the development of IH [24]. Our research demonstrated that there was a significant increase in the expression of PCNA and ClvCasp-3 within the intima-media layer of the vein graft, and it was prevented by CES. Thus, to be sure, the CES could effectively inhibit the proliferation and apoptosis of VSMCs. Unfortunately, we do not know which one is more important between proliferation and apoptosis. Phenotypic switching of VSMCs is described as functional and structural changes, which translate from the contractile phenotype to the dedifferentiated phenotype during the early phase after implantation. Contractile phenotype VSMCs maintain a quiescent state, in which they have a low rate of proliferation and synthetic activity and express various unique contractile proteins, such as SM22*α* and calponin [25]. After implantation, the VSMCs changed the phenotype to migrate into the intima from the medial layer and rapid proliferation to form IH [26]. Our study also observed that SM22*α* was significantly downregulated, and the CES partly preserved the normal vein phenotype as a way to inhibit the proliferation and apoptosis of VSMCs.

Although our research demonstrated that the CES could effectively mitigate IH and improved the remodeling of vein grafts, there were still several limitations in our study. First, this was a relatively short-term experiment with a limited follow-up period. Although the short-term beneficial effects are promising, it is uncertain whether the protective effects of the CES will persist for a longer time. Second, in our animal models, we used end-to-end anastomosis rather than end-to-side anastomosis, which was widely used in CABG, to observe the inhibitory action of the CES on IH of vein graft. Therefore, it is still unclear whether the CES exerts a similar effect in clinic. But, we still believe that the research provides a good basis for further research. Compared with rabbits, a porcine arteriovenous graft model would be more suitable for the observation of the long-term effect of the CES on the inhibition of IH. Thus, we plan to perform the further research in a porcine arteriovenous graft model to investigate the long-term effects of CES.

## 5. Conclusion

Our study provided morphological and molecular evidence that the CES is effective in mitigating IH and improving the molding of vein grafts in a rabbit arteriovenous graft model. The beneficial effects appear to be associated with marked reduction in the proliferation and apoptosis of VSMCs and partly preserving the phenotype of VSMCs. Although the short-term beneficial effects of the CES seem promising, the long-term protective effects of the CES on the adaptation process of vein grafts warrant further investigating.

## Figures and Tables

**Table 1:** Sequences of the polymerase chain reaction primers.

Gene	DNA sequence (5′ to 3′)
PCNA	Forward: GGACTTAGATGTTGAACAGCTTGG
	Reverse: TTCTCCACTGGCGGAAAACTT
ClvCasp-3	Forward: CAGACAGTGGGGTTGACTATGACA
	Reverse: AGCGTACTCTTTCAGCATGGCA
SM22*α*	Forward: TGACCAAGAATGATGGGCACTA
	Reverse: AACTCCCGCTTGTGCTCCT
GAPDH	Forward: AAGTGCGACGTGGACATCCG
	Reverse: GGGCGGTGATCTCCTTCTGC

PCNA: proliferating cell nuclear antigen; ClvCasp-3: active cleaved-caspase-3; SM22*α*: smooth muscle 22 alpha; GAPDH: glyceraldehyde-3-phosphate dehydrogenase.
